# Supplementary material for: The type VI secretion system governs strain maintenance in a wild mammalian gut microbiome
Source: eLife. 2026 Oct 2;15:RP110200. doi: 10.7554/eLife.110200 (PMC13633076; doi:10.7554/eLife.110200)
Supplement: Peer review history

# Editor report: eLife Assessment

**DOI:** 10.7554/eLife.110200.3.sa0

**Authors:** Caetano Antunes

**Affiliations:** University of Kansas United States

**Keywords:** Convincing, Fundamental

This **fundamental** work significantly advances our understanding of how contact-dependent antagonism enables keystone bacteria to establish and maintain their niche over time. The evidence obtained is **convincing**, supporting most of the conclusions drawn. This work will be of significant interest to the microbiome research community.

# Referee report: Reviewer #1 (Public review)

**DOI:** 10.7554/eLife.110200.3.sa1

Summary:

In this study, the authors investigate the physiological role of the Type VI secretion system (T6SS) in a naturally evolved gut microbiome derived from wild mice (the WildR microbiome). Focusing on *Bacteroides acidifaciens*, the authors use newly developed genetic tools and strain replacement strategies to test how T6SS-mediated antagonism influences colonization, persistence, and fitness within a complex gut community. They further show that the T6SS resides on an integrative and conjugative element (ICE), is distributed among select community members, and can be horizontally transferred, with context-dependent effects on colonization and persistence. The authors conclude that the T6SS stabilizes strain presence in the gut microbiome while imposing ecological and physiological constraints that shape its value across contexts.

This study is likely to have significant impact on the microbiome field by moving experimental tests of T6SS function out of simplified systems and into a naturally co-evolved gut community. The WildR system, together with the strain replacement strategy, ICE-seq approach, and genetic toolkit, represents a powerful and reusable platform for future mechanistic studies of microbial antagonism and mobile genetic elements in vivo.

The datasets-including isolate genomes, metagenomes, and ICE distribution maps-will be valuable community resources, particularly for researchers interested in strain-resolved dynamics, horizontal gene transfer, and ecological context dependence. Even where mechanistic resolution is incomplete, the work provides a strong experimental foundation upon which such questions can be directly addressed.

Overall, this study occupies a space between system building and mechanistic dissection. The authors demonstrate that the T6SS influences persistence and community structure in vivo, but the physiological basis of these effects remains unresolved. Interpreting the results as evidence of fitness costs or selective advantage therefore requires caution, as multiple ecological and host-mediated processes could produce similar abundance trajectories.

Placing the findings within the broader literature on microbial antagonism, particularly work emphasizing measurable costs, benefits, and tradeoffs, would help readers better contextualize what is directly demonstrated here versus what remains an open question. Viewed in this light, the principal contribution of the study is to show that such questions can now be addressed experimentally in a realistic gut ecosystem.

Strengths:

A major strength of this study is that it directly interrogates the physiological role of the T6SS in a naturally evolved gut microbiome, rather than relying on simplified pairwise or in vitro systems. By working within the WildR community, the authors advance beyond descriptive surveys of T6SS prevalence and address function in an ecologically relevant context.

The authors provide clear genetic evidence that *Bacteroides acidifaciens* uses a T6SS to antagonize co-resident Bacteroidales, and that loss of T6SS function specifically compromises long-term persistence without affecting initial colonization. This temporal separation is well designed and supports the conclusion that the T6SS contributes to maintenance rather than establishment within the community.

Another strength is the identification of the T6SS on an integrative and conjugative element (ICE) and the demonstration that this element is distributed among, and exchanged between, community members. The use of ICE-seq to track distribution and transfer provides strong support for horizontal mobility and adds mechanistic depth to the study.

Finally, the transfer of the T6SS-ICE into *Phocaeicola vulgatus* and the observation of context-dependent colonization benefits followed by decline is a compelling result that moves the study beyond simple "T6SS is beneficial" narratives and highlights ecological contingency.

Weaknesses:

Despite these strengths, there is a mismatch between the precision of the claims and the precision of the measurements, particularly regarding fitness costs, physiological burden, and mechanistic role of the T6SS.

First, while the authors conclude that the T6SS "stabilizes strain presence" and that its value is constrained by fitness costs, these costs are not directly measured. Persistence, abundance trajectories, and eventual loss are informative outcomes, but they do not uniquely identify fitness tradeoffs. Decline could arise from multiple non-exclusive mechanisms, including community restructuring, host-mediated effects, incompatibilities of the ICE in new hosts, or ecological retaliation, none of which are disentangled here.

Second, the manuscript frames the T6SS as having a defined physiological role, yet the data do not resolve which physiological processes are under selection. The experiments demonstrate that T6SS activity affects persistence, but they do not distinguish whether this occurs via direct killing, resource release, niche modification, or higher-order community effects. As a result, "physiological role" remains underspecified and risks being conflated with ecological outcome.

Third, although the authors emphasize context dependence, the study offers limited quantitative insight into what aspects of context matter. Differences between native and recipient hosts, or between early and late colonization phases, are described but not mechanistically interrogated, making it difficult to generalize beyond the specific cases examined.

Fourth is the lack of engagement with recent experimental literature demonstrating functional roles of the T6SS beyond simple interference competition. While the authors focus on persistence and competitive outcomes, they do not adequately situate their findings within recent work demonstrating that T6SS-mediated antagonism can serve additional physiological functions, including resource acquisition and DNA uptake, thereby linking killing to measurable benefits and tradeoffs. The absence of this literature makes it difficult to place the authors' conclusions about physiological role and fitness cost within the current conceptual framework of the field. Without this context, the physiological interpretation of the results remains incomplete, and alternative functional explanations for the observed dynamics are underexplored.

A further limitation concerns the taxonomic scope of the functional analysis. The authors state the role of the T6SS in the murine environment is functionally investigated using genetically tractable *Bacteroides* species, citing lack of genetic tools for *Mucispirillum schaedleri*. While this is a reasonable practical choice, it means that a substantial fraction of T6SS-encoding species in the WildR community are not experimentally interrogated. Consequently, conclusions about the role of the T6SS in the murine gut necessarily reflect the subset of taxa that are genetically accessible and may not fully capture community-level or niche-specific functions of T6SS activity. Given that *M. schaedleri* is represented as a metagenome-assembled genome, its isolation and genetic manipulation would be technically challenging. Nonetheless, explicitly acknowledging this limitation and slightly tempering claims of generality would strengthen the manuscript.

Finally, several interpretations would benefit from more cautious language. In particular, claims invoking fitness costs, selective advantage, or physiological burden should be explicitly framed as inferences from persistence dynamics, rather than as direct measurements, unless supported by additional quantitative fitness or growth assays.

Comments on revised version.

The authors have addressed my main concerns by more clearly distinguishing ecological outcomes from directly measured physiological mechanisms. They have moderated claims about fitness costs and benefits, replaced "physiological" with "ecological" where appropriate, expanded the discussion of potential downstream benefits of T6SS-mediated killing, and acknowledged the limited taxonomic scope of the functional analyses. The persistence trajectories support context-dependent relative fitness effects, although they do not identify the specific physiological basis of those effects. The revised manuscript now generally maintains this distinction. These revisions substantially improve the precision and balance of the manuscript.

# Referee report: Reviewer #2 (Public review)

**DOI:** 10.7554/eLife.110200.3.sa2

Summary:

In this study, the authors set out to determine how a contact-dependent bacterial antagonistic system contributes to the ability of specific bacterial strains to persist within a complex, native gut community derived from wild animals. Rather than focusing on simplified or artificial models, the authors aimed to examine this system in a biologically realistic setting that captures the ecological complexity of the gut environment. To achieve this, they combined controlled laboratory experiments with animal colonization studies and sequencing-based tracking approaches that allow individual strains and mobile genetic elements to be followed over time.

Strengths:

A major strength of the work is the integration of multiple complementary approaches to address the same biological question. The use of defined but complex communities, together with in vivo experiments, provides a strong ecological context for interpreting the results. The data consistently show that the antagonistic system is not required for initial establishment but plays a critical role in long-term strain persistence, an insight that moves beyond traditional invasion-based views of microbial competition. The observation that transferable genetic elements can confer only temporary advantages, and may impose longer-term costs depending on community context, adds important nuance to current understanding of microbial fitness.

Weaknesses:

Overall, the study is not a lack of evidence, but a deliberate trade-off between ecological realism and mechanistic resolution, which leaves some causal pathways open to interpretation.

Comments on revised version.

The authors have addressed all previous concerns thoroughly and satisfactorily.

# Referee report: Reviewer #3 (Public review)

**DOI:** 10.7554/eLife.110200.3.sa3

Summary:

In this work, the authors investigate the contribution of the type VI secretion system of Bacteroidales to gut microbiome assembly and the targeting of closely related species. They demonstrate that *B. acidifaciens* relies on T6SS-mediated antagonism to prevent displacement by co-resident Bacteroidales and other members of the microbiome, allowing it to persist in the gut. They also developed new tools for analyzing the distribution of mobile genetic elements. This study advances our understanding of how molecular systems contribute to shaping complex microbial communities.

Strengths:

The use of a gnotobiotic model colonized with a wild-mouse microbiome is a significant strength of this study. This approach allows tracking of microbiome changes over time and evaluating the targeting by Bacteroidales carrying T6SS in a more natural setting. The development of ICE-seq for mapping the distribution of the T6SS in the microbiome is remarkable, enabling the study of how this bacterial weapon is transferred between microbiome members without requiring long-read metagenomics methods.

Weaknesses:

Some conclusions are based on a limited number of mice per condition. This could be due to the complexity of using a gnotobiotic mouse model, but this should be considered when interpreting the data.

Overall, the authors successfully achieved their objectives, and their experimental design and results support their findings. As mentioned in the discussion, it would be important to investigate the role of the T6SS in resilience to microbiome disturbances, such as antibiotics, diet, or pathogen invasion. This work represents a step forward in understanding how contact-dependent competition influences the gut microbiome in relevant ecological contexts.

# Author comment: Author response

**DOI:** 10.7554/eLife.110200.3.sa4

**Authors:** Beth A Shen, Kyle L Asfahl, Bentley Lim, Savannah K Bertolli, Samuel S Minot, Matthew C Radey, Kelsi M Penewit, Billy Ngo, Stephen J Salipante, Christopher D Johnston, S Brook Peterson, Andrew L Goodman, Joseph D Mougous

**Affiliations:** University of Wisconsin-Madison Madison United States; University of Washington Seattle United States; Yale University New Haven United States; University of Washington Seattle United States; Fred Hutchinson Cancer Center Seattle United States; University of Washington Seattle United States; University of Washington Seattle United States; University of Washington Seattle United States; University of Washington Seattle United States; The University of Texas MD Anderson Cancer Center Houston United States; University of Washington Seattle United States; Yale University New Haven United States; University of Washington Seattle United States

The following is the authors’ response to the original reviews.

We appreciate that the reviewers provided an overall positive assessment of our manuscript and offered constructive suggestions for improvement. All three reviewers noted that a key strength of our study is the implementation of a gut microbiome model for the characterization of interbacterial antagonism pathways such as the type VI secretion system (T6SS) that approaches natural complexity. They note our work represents a significant advance in microbiome research, and generates resources that will be of use to many researchers in the field. Two of the reviewers point out that the complexity of our model limits the nature of measurements we can make, and suggest we temper the strength of the some of the conclusions we draw. As noted in more detail below, in our revised manuscript, we have used more precise wording to characterize our findings, and we are more explicit about the connection between the measurements we made and what we can conclude about the physiological role of the T6SS in the gut microbiome.

**Public Reviews:**

**Reviewer #1 (Public review):**

Summary:

In this study, the authors investigate the physiological role of the Type VI secretion system (T6SS) in a naturally evolved gut microbiome derived from wild mice (the WildR microbiome). Focusing on *Bacteroides acidifaciens*, the authors use newly developed genetic tools and strain-replacement strategies to test how T6SS-mediated antagonism influences colonization, persistence, and fitness within a complex gut community. They further show that the T6SS resides on an integrative and conjugative element (ICE), is distributed among select community members, and can be horizontally transferred, with context-dependent effects on colonization and persistence. The authors conclude that the T6SS stabilizes strain presence in the gut microbiome while imposing ecological and physiological constraints that shape its value across contexts.

This study is likely to have a significant impact on the microbiome field by moving experimental tests of T6SS function out of simplified systems and into a naturally coevolved gut community. The WildR system, together with the strain replacement strategy, ICE-seq approach, and genetic toolkit, represents a powerful and reusable platform for future mechanistic studies of microbial antagonism and mobile genetic elements in vivo.

The datasets, including isolate genomes, metagenomes, and ICE distribution maps, will be a valuable community resource, particularly for researchers interested in strainresolved dynamics, horizontal gene transfer, and ecological context dependence. Even where mechanistic resolution is incomplete, the work provides a strong experimental foundation upon which such questions can be directly addressed.

Overall, this study occupies a space between system building and mechanistic dissection. The authors demonstrate that the T6SS influences persistence and community structure in vivo, but the physiological basis of these effects remains unresolved. Interpreting the results as evidence of fitness costs or selective advantage, therefore, requires caution, as multiple ecological and host-mediated processes could produce similar abundance trajectories.

Placing the findings within the broader literature on microbial antagonism, particularly work emphasizing measurable costs, benefits, and tradeoffs, would help readers better contextualize what is directly demonstrated here versus what remains an open question. Viewed in this light, the principal contribution of the study is to show that such questions can now be addressed experimentally in a realistic gut ecosystem.

We thank the reviewer for this thoughtful summary of our study. We were glad to read they conclude our work will have a significant impact on the microbiome field and that the resources we have developed will be of value to the community.

Strengths:

A major strength of this study is that it directly interrogates the physiological role of the T6SS in a naturally evolved gut microbiome, rather than relying on simplified pairwise or in vitro systems. By working within the WildR community, the authors advance beyond descriptive surveys of T6SS prevalence and address function in an ecologically relevant context.

The authors provide clear genetic evidence that *Bacteroides acidifaciens* uses a T6SS to antagonize co-resident Bacteroidales, and that loss of T6SS function specifically compromises long-term persistence without affecting initial colonization. This temporal separation is well designed and supports the conclusion that the T6SS contributes to maintenance rather than establishment within the community.

Another strength is the identification of the T6SS on an integrative and conjugative element (ICE) and the demonstration that this element is distributed among, and exchanged between, community members. The use of ICE-seq to track distribution and transfer provides strong support for horizontal mobility and adds mechanistic depth to the study.

Finally, the transfer of the T6SS-ICE into *Phocaeicola vulgatus* and the observation of context-dependent colonization benefits followed by decline is a compelling result that moves the study beyond simple "T6SS is beneficial" narratives and highlights ecological contingency.

We appreciate this detailed and nuanced characterization of the strengths of our study.

Weaknesses:

Despite these strengths, there is a mismatch between the precision of the claims and the precision of the measurements, particularly regarding fitness costs, physiological burden, and the mechanistic role of the T6SS.

We acknowledge that in some places, our manuscript could benefit from greater precision in the language we use when linking the outcomes we observe in our study to their potential underlying causes. Specific revisions we made to address this concern are described below.

First, while the authors conclude that the T6SS "stabilizes strain presence" and that its value is constrained by fitness costs, these costs are not directly measured. Persistence, abundance trajectories, and eventual loss are informative outcomes, but they do not uniquely identify fitness tradeoffs. Decline could arise from multiple nonexclusive mechanisms, including community restructuring, host-mediated effects, incompatibilities of the ICE in new hosts, or ecological retaliation, none of which are disentangled here.

We agree that multiple mechanisms could explain why populations of certain species carrying a T6SS decline over time, and why for others, the T6SS contributes to long-term persistence. Our use of the term “fitness cost” to describe the phenomenon of decline observed for *P. vulgatus* carrying the T6SS was not meant to imply any particular underlying mechanism, but was rather our attempt to characterize the phenotypic outcome we observed in simplified terms. We note that ecological context is an important determinant of the fitness cost or benefit of any given trait, and our study sheds light on the importance of the presence of the WildR community and the mouse intestinal environment to the fitness contribution of the T6SS to *B. acidifaciens* and *P. vulgatus*. Nonetheless, to avoid implying an overly simplistic interpretation of our results, we have modified our language in the manuscript in several places when describing the role of the T6SS in species persistence in mice colonized with the WildR community.

Second, the manuscript frames the T6SS as having a defined physiological role, yet the data do not resolve which physiological processes are under selection. The experiments demonstrate that T6SS activity affects persistence, but they do not distinguish whether this occurs via direct killing, resource release, niche modification, or higher-order community effects. As a result, "physiological role" remains underspecified and risks being conflated with ecological outcome.

We acknowledge that our study does not fully resolve the physiological processes under selection that mediate role of the T6SS in maintaining *B. acidifaciens* populations in WildR-colonized mice. Indeed, several of the outcomes of T6SS activity the reviewer lists, such as target cell killing and nutrient release, are inextricably linked and thus inherently difficult to disentangle. We note that we did attempt to measure higher-order community effects of T6SS activity with metagenomic sequencing, but acknowledge that this approach may not have been sufficiently sensitive to detect small community shifts mediated by a relatively low-abundance species. To address the concern that our current framing implies more of a mechanistic understanding that our study achieves, we have substituted “ecological” for “physiological” where appropriate throughout the manuscript.

Third, although the authors emphasize context dependence, the study offers limited quantitative insight into what aspects of context matter. Differences between native and recipient hosts, or between early and late colonization phases, are described but not mechanistically interrogated, making it difficult to generalize beyond the specific cases examined.

We are not entirely clear what the reviewer means by “differences between native and recipient hosts”, but we agree that additional quantitative studies will be needed to address the generalizability of our findings. Future studies are also needed to address the mechanistic basis for the difference in the benefit conferred by the T6SS that we observed between *P. vulgatus* and *B. acidifaciens*.

Fourth is the lack of engagement with recent experimental literature demonstrating functional roles of the T6SS beyond simple interference competition. While the authors focus on persistence and competitive outcomes, they do not adequately situate their findings within recent work demonstrating that T6SS-mediated antagonism can serve additional physiological functions, including resource acquisition and DNA uptake, thereby linking killing to measurable benefits and tradeoffs. The absence of this literature makes it difficult to place the authors' conclusions about physiological role and fitness cost within the current conceptual framework of the field. Without this context, the physiological interpretation of the results remains incomplete, and alternative functional explanations for the observed dynamics are underexplored.

We thank the reviewer for specifically highlighting the potential pertinence of this literature to our study. Indeed, we did not cite studies indicating a link between T6SS activity and the uptake of DNA and other resources released by targeted cells. As we note above, the release of intracellular contents from target cells is an inevitable consequence of the delivery of lytic effectors. Thus, distinguishing between fitness benefits conferred from the elimination of competitor species and those arising from scavenging the nutrients released during this process is not straightforward. Measuring the benefits deriving from the uptake of certain released molecules, such as DNA, was not immediately feasible in the system employed in this study and instead we focused on the direct lytic consequences of the effectors delivered via the T6SS. We revised the Discussion to include reference to these possible downstream benefits of T6SS activity (Lines 476-479).

A further limitation concerns the taxonomic scope of the functional analysis. The authors state that the role of the T6SS in the murine environment is functionally investigated using genetically tractable *Bacteroides* species, citing the lack of genetic tools for *Mucispirillum schaedleri*. While this is a reasonable, practical choice, it means that a substantial fraction of T6SS-encoding species in the WildR community are not experimentally interrogated. Consequently, conclusions about the role of the T6SS in the murine gut necessarily reflect the subset of taxa that are genetically accessible and may not fully capture community-level or niche-specific functions of T6SS activity. Given that *M. schaedleri* is represented as a metagenome-assembled genome, its isolation and genetic manipulation would be technically challenging. Nonetheless, explicitly acknowledging this limitation and slightly tempering claims of generality would strengthen the manuscript.

The reviewer points out that studying the T6SS activity in *M. schadleri* would potentially expand the generality of our claims. We agree that having an isolate of this species along with genetic tools for its manipulation would allow us to probe the importance of the T6SS in the gut microbiome more broadly. At the suggestion of the reviewer, we have added explicit mention of the potential benefit of studying the T6SS in this organism to the Discussion (lines 538-539), an endeavor that lies outside of the scope of the current study.

Finally, several interpretations would benefit from more cautious language. In particular, claims invoking fitness costs, selective advantage, or physiological burden should be explicitly framed as inferences from persistence dynamics, rather than as direct measurements, unless supported by additional quantitative fitness or growth assays.

We agree with the reviewer that invoking fitness costs, selective advantages or physiological burdens should be done cautiously, and have made revisions to our manuscript where we acknowledge that more precise language was needed (line 43, 416, line 417). However, we would also argue invoking fitness costs and benefits when describe strain persistence dynamics in mice has substantial precedent in the literature (Feng et al. 2020, Brown et al. 2021, Park et al. 2022, Segura Munoz et al. 2022), to list a handful of representative examples published by different groups. It is unclear to us what additional in vivo growth measurements could be taken to substantiate our claim that the T6SS provides a fitness benefit to *B. acidifaciens* during prolonged gut colonization, or that carrying the ICE imposes a fitness cost on *P. vulgatus* during longterm colonization. Our in vitro experiments evaluating the competitiveness conferred by T6SS activity provide a measure of insight into its fitness benefits, but as our in vivo strain persistence data and the work of many others show, in vitro measurements do not necessarily capture in vivo parameters.

**Reviewer #2 (Public review):**

Summary:

In this study, the authors set out to determine how a contact-dependent bacterial antagonistic system contributes to the ability of specific bacterial strains to persist within a complex, native gut community derived from wild animals. Rather than focusing on simplified or artificial models, the authors aimed to examine this system in a biologically realistic setting that captures the ecological complexity of the gut environment. To achieve this, they combined controlled laboratory experiments with animal colonization studies and sequencing-based tracking approaches that allow individual strains and mobile genetic elements to be followed over time.

Strengths:

A major strength of the work is the integration of multiple complementary approaches to address the same biological question. The use of defined but complex communities, together with in vivo experiments, provides a strong ecological context for interpreting the results. The data consistently show that the antagonistic system is not required for initial establishment but plays a critical role in long-term strain persistence. This insight that moves beyond traditional invasion-based views of microbial competition. The observation that transferable genetic elements can confer only temporary advantages, and may impose longer-term costs depending on community context, adds important nuance to current understanding of microbial fitness.

We thank the reviewer for the positive feedback and are glad they agree our study provides new insight into the role of interbacterial antagonism in natural communities.

Weaknesses:

Overall, there is not a lack of evidence, but a deliberate trade-off between ecological realism and mechanistic resolution, which leaves some causal pathways open to interpretation.

The reviewer makes a good point that the complexity of the experimental system we employ precludes some lines of experimentation that would yield more mechanistic information. As the reviewer notes, we were aware of the tradeoff between mechanistic resolution and ecological realism when selecting our experimental system. Our deliberate choice to favor biological complexity over mechanistic clarity in this study stemmed from our perception that a major gap in understanding of the T6SS and other antagonism pathways lies in defining their ecological function in complex microbial communities.

**Reviewer #3 (Public review):**

Summary:

Shen et al. investigate the contribution of the type VI secretion system of Bacteroidales in the gut microbiome assembly and targeting of closely related species. They demonstrate that *B. acidifaciens* relies on T6SS-mediated antagonism to prevent displacement by co-resident Bacteroidales and other members of the microbiome, allowing *B. acidifaciens* to persist in the gut.

Strengths:

Using a gnotobiotic model colonized with a wild-mouse microbiome is a significant strength of this study. This approach allows tracking of microbiome changes over time and directly examining targeting by Bacteroidales carrying T6SS in a more natural setting. The development of ICE-seq for mapping the distribution of the T6SS in the microbiome is remarkable, enabling the study of how this bacterial weapon is transferred between microbiome members without requiring long-read metagenomics methods.

We thank the reviewer for their enthusiasm toward our study.

Weaknesses:

Some conclusions are based on only four mice per condition. The author should consider increasing the sample size.

We agree that in some experiments it would be beneficial to increase the sample size from four mice. However, the experiments we performed for this study are time and resource-intensive. Additionally, the experiments on which we base our primary conclusions were all independently replicated with similar results. Given these factors, we determined that the extra confidence that might be afforded by increasing our sample size did not merit the delay in publication and investment in resources that would be required.

Overall, the authors successfully achieved their objectives, and their experimental design and results support their findings. As mentioned in the discussion, it would be important to investigate the role of the T6SS in resilience to disturbances in the microbiome, such as antibiotics, diet, or pathogen invasion. This work represents a step forward in understanding how contact-dependent competition influences the gut microbiome in relevant ecological contexts.

We agree that investigating the role of the T6SS during perturbations of the microbiome is a key next step for this work and thank the reviewer for highlighting this important future direction.

**Recommendations for the authors:**

**Reviewer #2 (Recommendations for the authors):**

Beth A. Shen et al. present a comprehensive and carefully executed study investigating the ecological role of the type VI secretion system (T6SS) in maintaining bacterial strains within a native, complex gut microbiome derived from wild mice. By integrating genetic manipulation, metagenomic and sequencing-based tracking approaches, in vitro competition assays, and gnotobiotic colonization experiments, the authors provide compelling evidence that the T6SS functions primarily as a persistence factor rather than a determinant of initial colonization.

The study is conceptually strong and addresses an important gap in our understanding of how interbacterial antagonistic systems operate in complex, native microbial communities. The manuscript is generally well organized, the data are clearly presented, and the main conclusions are supported by robust experimental evidence. In particular, the demonstration that T6SS-encoding ICEs confer context- and hostdependent fitness effects, including transient benefits and potential long-term costs, adds important nuance to prevailing models of microbial competition.

That said, several aspects of the study would benefit from clarification and deeper mechanistic discussion. Addressing the points below would further strengthen the rigor and interpretability of the work. Overall, this is a strong and interesting manuscript, requiring some revisions.

We greatly appreciate the positive summary of our work by the reviewer, which highlights the multi-faceted approach we took to address gaps in our understanding of interbacterial antagonism in the microbiome. It is our hope that the reviewer agrees that our revisions of the manuscript, based on their feedback, clarify our methods and strengthen the interpretability of our work.

Major comments

(1) The competition assays in Figure 2B suggest that T6SS-dependent fitness effects are most pronounced among members of the order Bacteroidales. However, these experiments primarily measure population-level competitive outcomes rather than direct T6SS-mediated targeting events. In addition, the limited number of non-Bacteroidales strains included in the assay makes it difficult to conclude that T6SS activity is strictly restricted to closely related taxa.

The authors should either temper their conclusions regarding target specificity or clarify that these data reflect competitive outcomes rather than direct evidence of targeting. Expanding the discussion to acknowledge these limitations would improve interpretative accuracy.

With regards to the measurement we employed for assessing T6SS-mediated targeting, we acknowledge that this is, to a degree, an indirect way of determining T6SS targeting. However, there is extensive precedent in the literature for the use of similar assays in assessing targeting by many contact-dependent antagonism systems including the T6SS in Bacteroidales (Russell et al. 2014, Chatzidaki-Livanis et al. 2016, Wexler et al. 2016) and many Proteobacteria (e.g. (Hood et al. 2010)), the T4SS in *Xanthomonas citri* (Souza et al. 2015), the CDI system in *Escherichia coli* (Aoki et al. 2005), and the Esx system in *Streptococcus intermedius* (Whitney et al. 2017). In these studies, targeting was demonstrated by specific depletion of the competitor strain in the presence of a strain encoding an active antagonism system. We acknowledge that the competitive index we report Figure 2B reflects the relative population levels of both species in the assay, and thus does not directly show target species depletion. We opted to use this metric to display the data in the manuscript as a way of efficiently encapsulating and comparing many strain combinations in a single figure, and because the competitive index differences we observed in these derive from differences in target species growth yields (see Author response image 1, indicating growth yields from a representative strain pairing).

**Author response image 1.** The T6SS of *B. acidifaciens* targets a WildR-derived *P. vulgatus* strain. CFUs indicate populations of the indicated strains after co-culture of wild-type or T6SS-inactivated *B. acidifaciens* with *P. vulgatus*. Data represent means and standard errors (n=3, *P<0.01, t-test with log ><0.01, t- test with log transformed data)

We additionally acknowledge that more extensive testing is needed to fully understand the target range of the Bacteroidales T6SS. In our study, we assessed targeting of every WildR species that was readily culturable, which to the best of our knowledge, represents the broadest panel of targets for the Bacteroidales T6SS to be tested to date. We limited our testing to these strains, as the goal of these experiments was to gain insight into which co-residents of the WildR could be targeted by *B. acidifaciens*. We agree that testing of a broader cross-section of potential targets has merits, but this would require targeted cultivation strategies to obtain these organisms, and lies outside the scope of the current study. We have revised the manuscript to clarify that the target range testing encompassed the diversity of isolates available (p. 10, lines 231-236).

(2) The bae1 gene encoded in *Bacteroides caecimuris* F12 contains a frameshift mutation. It would be valuable for the authors to comment on whether such frameshift mutations are a common genomic feature among gut-associated *Bacteroides* species in murine models. In addition, comparative analysis of human gut metagenomic datasets could reveal whether homologous effector proteins are present in commensal *Bacteroides* populations, and whether these homologs exhibit similar disruptive mutations.

More broadly, the manuscript would benefit from a discussion of whether expression of a fully functional bae1 effector might impose a fitness cost on Bacteroidales members, for example, through metabolic burden or altered resource allocation. This is particularly relevant in light of recent studies demonstrating that T6SS effectors can drive physiological trade-offs by modulating metabolic dynamics (PMID: 40592326). Integrating this perspective would strengthen the evolutionary interpretation of effector mutagenesis.

We agree with the reviewer that the functional and evolutionary significance of the point mutation in *bae1* merits further investigation. Following the reviewer's suggestion, we looked in our own datasets and available public datasets from mouse and human microbiomes for evidence of *bae1* inactivation. Unfortunately, the gene is present at a low enough frequency that these analyses were inconclusive. In our own metagenomic data from WildR mice, we did not obtain sufficient sequencing depth to assess the frequency at which *bae1* is inactivated across genomes. We found a single complete copy of *bae1* identical to that of *B. acidifaciens* in one published mouse microbiome-derived MAG, and detected fragments of the gene in a number of publicly available isolate and MAG genomes, but these were too low of quality to assess whether or not the gene was intact.

As to whether or not *bae1* expression imposes a fitness cost in the producing organism, we think this is unlikely to be significant, given that the impacts of Bae1 will be neutralized by the accompanying immunity protein. We speculate that the point mutation in the *B. caecimuris* gene is more likely to have arisen through genetic drift than as a result of selection.

(3) Quantification and tracking of ICE transfer in vivo. In Figure 4D, the authors assess the abundance of resident *P. vulgatus* populations in germ-free mice co-gavaged with wild-type strains and derivatives carrying either the intact ICE or ICE ΔtssC. Because both ICE variants are capable of horizontal transfer, it is essential to clearly describe how the authors distinguish between (i) the original wild-type strain, (ii) engineered donor strains, and (iii) recipient strains that have newly acquired the ICE or ICE ΔtssC.

Clarification of the specific molecular or sequencing-based strategies used to discriminate these populations is necessary to ensure accurate interpretation of the colonization dynamics.

In this experiment, the *P. vulgatus* strains we introduced which carried the ICE (either the wild-type version or ICE D*tssC*) also contained an erythromycin resistance cassette (*ermG*) inserted distal to the ICE insertion site. Populations of the ICE-containing strain were quantified by either qPCR targeting the *ermG* gene (Fig. 4D, Supplemental Fig. 4D) or by plating on erythromycin-containing media (Fig. 4F). Endogenous *P. vulgatus* populations were quantified by qPCR targeting the *ermG* insertion site, which is disrupted in the marked strain. These methodological details have been added to the figure legend for clarity. We acknowledge that transfer of the ICE between introduced and endogenous populations is possible, and would not be detected by these metrics. To assess whether this occurs, we performed ICE-seq analyses on samples collected from mice colonized by the WildR and *P. vulgatus* ICE at early (7 days) and late (56 days) time points. These analyses revealed that overall, ICE distribution in this experiment was similar to that observed in mice colonized with the WildR alone (Figure 4A and Author response image 2). They additionally provided corroborating evidence that the population of ICE-containing *P. vulgatus* declined over the course of the experiment. Importantly, the only ICE insertion site we detected in *P. vulgatus* in these samples was that found in the introduced *P. vulgatus* strain. Previous studies show that GA1-containing ICE can insert at numerous locations in *Bacteroides* sp. genomes, a finding supported by our mapping of the ICE insertion sites from in vitro transfer experiments (Supplemental Fig. 4C) (Garcia-Bayona et al. 2021). Thus, our ICE-seq detection of a sole *P. vulgatus* ICE insertion site indicates that transfer of the element between *P. vulgatus* populations is likely not occurring in our experiments.

**Author response image 2.** ICE-seq analysis indicates that introduction of *P. vulgatus* ICE into WildR-colonized mice has little impact on ICE distribution among endogenous strains. Graphs show frequency of mapped ICE junctions deriving from the indicated species as determined by 5¢ or 3¢ ICE-Seq analysis of DNA extracted from fecal samples collected either 7 or 56 days post-gavage of the WildR and *P. vulgatus* ICE into germ-free mice.

(4) The analysis of fitness trade-offs associated with ICE acquisition in *P. vulgatus* convincingly demonstrates that the benefits of ICE transfer are transient and contextdependent. However, the mechanistic basis of these trade-offs remains underexplored. While the study primarily attributes both benefits and costs to T6SS-mediated antagonism, the ICE likely encodes additional genes that could influence metabolism, regulation, or stress responses.

We agree with the reviewer that there are many mechanistic questions remaining regarding the benefits and costs associated with ICE acquisition, and acknowledge that we have not investigated the fitness contributions of ICE-encoded genes other than the T6SS. Indeed, as we noted in our discussion of the results from introducing *P. vulgatus* carrying the ICE into WildR-carrying mice, our data suggest that ICE genes outside the T6SS may be beneficial (lines 463-465). At the reviewer’s suggestion, we have reiterated the importance of considering the fitness contribution of genes beyond the T6SS in determining ICE distribution in the WildR community (line 527).

Minor comments:

(1) In lines 319 and 333, the manuscript refers to "Supplemental Figure 3F" and "Supplemental Figure 3G," respectively. However, the provided Supplemental Figure 3 appears to end at panel E. Please clarify or correct these references.

We have modified the text to reference the correct figure panels.

(2) Line 1043: The notation for "OD600" should be corrected for consistency and accuracy.

The notation for OD600 has been updated to be consistent throughout the manuscript.

**Reviewer #3 (Recommendations for the authors):**

Minor comments:

(1) Line 144. I would be careful of using "strong correlation, in this sentence. Although it shows a higher correlation than lab mice. Also, the labels in Figure 1A for mouse WildRF7 are confusing and not well explained in the figure legend.

We modified line 147 (new line in edited manuscript) to say “positive correlation” rather than “strong correlation” to better represent the result. We also revised the legend for Figure 1A to better explain the samples of WildR F7 that were analyzed.

(2) Line 155. It's unclear which strains were isolated from the WildR community, and the reason for isolating only 15 strains. Also, Supplemental Figure 1 shows 17 isolates, not 15.

We apologize for the confusion here. We isolated 17 strains, which is the number of distinct strains we were able to readily culture from this community. We obtained genome sequences for 15 of these, and were able to assemble a genome for one more of the strains from metagenomic data.

(3) Line 236. Is it known what makes *B. uniformis* resistant to *B. acidifaciens* carrying a T6SSS? Does it have an orphan immunity protein?

We do not know why *B. uniformis* is not targeted by *B. acidifaciens* under the conditions of our experiments. It does not encode homologs of the immunity genes *bai1* or *bai2*, and does not appear to be intrinsically resistant to targeting by this T6SS given that it is effectively targeted by *P. vulgatus* carrying the ICE (Fig.4b).

(4) Line 295. There is a consistent decline in C. acid abundance after 27 days in Figures 3B and 3C. How do you explain this? Is the endogenous B. acid expanding to outcompete C. acid exo since the total C. acid exo remains constant when gavaging 100x B. acid exo?

We believe that the eventual decline in the introduced population of *B. acidifaciens* is likely due to a fitness cost imposed by the erm resistance marker we employed. We noted this phenomenon when describing the results depicted in Fig. 3F-H, but neglected to include this explanation earlier. This oversight has been corrected (lines 315-317).

References

Aoki, S. K., R. Pamma, A. D. Hernday, J. E. Bickham, B. A. Braaten and D. A. Low (2005). "Contact-dependent inhibition of growth in *Escherichia coli*." Science 309(5738): 1245–1248.

Brown, E. M., H. Arellano-Santoyo, E. R. Temple, Z. A. Costliow, M. Pichaud, A. B. Hall, K. Liu, M. A. Durney, X. Gu, D. R. Plichta, C. A. Clish, J. A. Porter, H. Vlamakis and R. J. Xavier (2021). "Gut microbiome ADP-ribosyltransferases are widespread phage-encoded fitness factors." Cell Host Microbe 29(9): 1351-1365 e1311.

Chatzidaki-Livanis, M., N. Geva-Zatorsky and L. E. Comstock (2016). "*Bacteroides* fragilis type VI secretion systems use novel effector and immunity proteins to antagonize human gut Bacteroidales species." Proc Natl Acad Sci U S A 113(13): 3627– 3632.

Feng, L., A. S. Raman, M. C. Hibberd, J. Cheng, N. W. Griffin, Y. Peng, S. A. Leyn, D. A. Rodionov, A. L. Osterman and J. I. Gordon (2020). "Identifying determinants of bacterial fitness in a model of human gut microbial succession." Proc Natl Acad Sci U S A 117(5): 2622-2633.

Garcia-Bayona, L., M. J. Coyne and L. E. Comstock (2021). "Mobile Type VI secretion system loci of the gut Bacteroidales display extensive intra-ecosystem transfer, multispecies spread and geographical clustering." PLoS Genet 17(4): e1009541.

Hood, R. D., P. Singh, F. Hsu, T. Guvener, M. A. Carl, R. R. Trinidad, J. M. Silverman, B. B. Ohlson, K. G. Hicks, R. L. Plemel, M. Li, S. Schwarz, W. Y. Wang, A. J. Merz, D. R. Goodlett and J. D. Mougous (2010). "A type VI secretion system of *Pseudomonas aeruginosa* targets a toxin to bacteria." Cell Host Microbe 7(1): 25–37.

Park, S. Y., C. Rao, K. Z. Coyte, G. A. Kuziel, Y. Zhang, W. Huang, E. A. Franzosa, J. K. Weng, C. Huttenhower and S. Rakoff-Nahoum (2022). "Strain-level fitness in the gut microbiome is an emergent property of glycans and a single metabolite." Cell 185(3): 513-529 e521.

Russell, A. B., A. G. Wexler, B. N. Harding, J. C. Whitney, A. J. Bohn, Y. A. Goo, B. Q. Tran, N. A. Barry, H. Zheng, S. B. Peterson, S. Chou, T. Gonen, D. R. Goodlett, A. L. Goodman and J. D. Mougous (2014). "A type VI secretion-related pathway in *Bacteroidetes* mediates interbacterial antagonism." Cell Host Microbe 16(2): 227–236.

Segura Munoz, R. R., S. Mantz, I. Martinez, F. Li, R. J. Schmaltz, N. A. Pudlo, K. Urs, E. C. Martens, J. Walter and A. E. Ramer-Tait (2022). "Experimental evaluation of ecological principles to understand and modulate the outcome of bacterial strain competition in gut microbiomes." ISME J 16(6): 1594-1604.

Souza, D. P., G. U. Oka, C. E. Alvarez-Martinez, A. W. Bisson-Filho, G. Dunger, L. Hobeika, N. S. Cavalcante, M. C. Alegria, L. R. Barbosa, R. K. Salinas, C. R. Guzzo and C. S. Farah (2015). "Bacterial killing via a type IV secretion system." Nat Commun 6: 6453.

Wexler, A. G., Y. Bao, J. C. Whitney, L. M. Bobay, J. B. Xavier, W. B. Schofield, N. A. Barry, A. B. Russell, B. Q. Tran, Y. A. Goo, D. R. Goodlett, H. Ochman, J. D. Mougous and A. L. Goodman (2016). "Human symbionts inject and neutralize antibacterial toxins to persist in the gut." Proc Natl Acad Sci 113(13): 3639–3644.

Whitney, J. C., S. B. Peterson, J. Kim, M. Pazos, A. J. Verster, M. C. Radey, H. D. Kulasekara, M. Q. Ching, N. P. Bullen, D. Bryant, Y. A. Goo, M. G. Surette, E. Borenstein, W. Vollmer and J. D. Mougous (2017). "A broadly distributed toxin family mediates contact-dependent antagonism between gram-positive bacteria." Elife 6(Jul 11): e26938.
